# Supplementary material for: Characterization of the mbsA Gene Encoding a Putative APSES Transcription Factor in Aspergillus fumigatus
Source: Int J Mol Sci. 2021 Apr 6;22(7):3777. doi: 10.3390/ijms22073777 (PMC8038847; doi:10.3390/ijms22073777)
Supplement: Supplementary file 1 [file ijms-22-03777-s001.pdf]

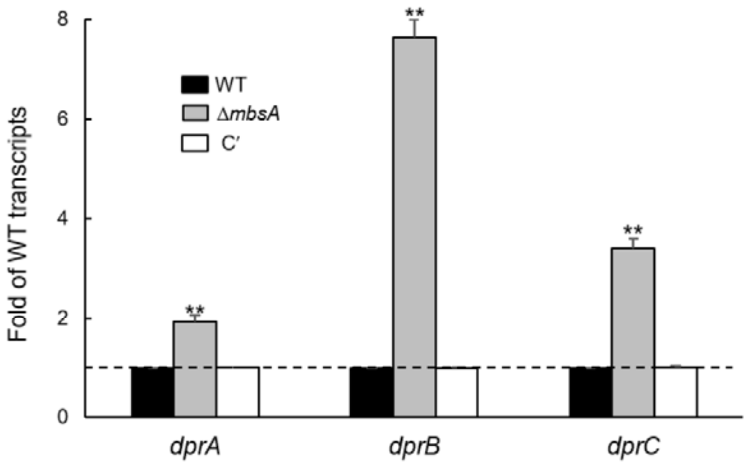

Supplementary Figure S1. Expression of *dpr* genes. RT-qPCR analysis of genes coding dehydrin-like proteins in WT, Δ*mbsA*, and C′ strains. The *ef1α* gene as the endogenous control. Statistical differences between strains were evaluated with ANOVA test: $**p < 0.01$.

Table S1. Differentially expressed toxin related genes in Δ*mbsA* relative to WT strain.

ID	Gene symbol	Annotation	Fold change	*p*-value
8112	AFUA_6G09730	cytochrome P450 oxidoreductase GliF	168.13	0.015
8110	AFUA_6G09710	MFS gliotoxin efflux transporter GliA	109.36	0.005
8113	AFUA_6G09740	thioredoxin reductase GliT	60.74	0.005
8108	AFUA_6G09690	glutathione S-transferase GliG	26.02	0.024
8109	AFUA_6G09700	gliotoxin biosynthesis protein GliK	21.88	0.011
8107	AFUA_6G09680	O-methyltransferase GliM	17.57	0.020
4619	AFUA_3G14710	toxin biosynthesis protein (Fum3), putative	14.43	0.043
8114	AFUA_6G09745	conserved hypothetical protein	12.91	0.023
4710	AFUA_4G00400	O-methyltransferase, putative	12.31	0.003
108	AFUA_1G01450	toxin biosynthesis protein, putative	11.94	0.001
8111	AFUA_6G09720	methyltransferase GliN	9.83	0.094
4440	AFUA_3G12910	O-methyltransferase GliM-like, putative	7.75	0.040
8106	AFUA_6G09670	cytochrome P450 oxidoreductase GliC	7.12	0.003
6174	AFUA_5G02640	O-methyltransferase, putative	6.45	0
8105	AFUA_6G09660	nonribosomal peptide synthase GliP	6.43	0.006
8103	AFUA_6G09640	aminotransferase GliI	5.54	0.037
5867	AFUA_4G14240	O-methyltransferase, putative	3.33	0.011
6847	AFUA_5G10320	toxin biosynthesis protein (GliH), putative	3.25	0.010
3266	AFUA_2G17820	O-methyltransferase	2.59	0.010
6846	AFUA_5G10310	AflR-like C6 zinc cluster transcription factor, putative	2.44	0.266
4834	AFUA_4G02730	O-methylsterigmatocystin oxidoreductase, putative	1.68	0.001
5888	AFUA_4G14520	toxin biosynthesis protein, putative	1.35	0.043
5890	AFUA_4G14540	C6 transcription factor (AflR), putative	0.94	0.087
2071	AFUA_2G04380	LaeA-like methyltransferase, putative	0.70	0.085
1335	AFUA_1G14660	regulator of secondary metabolism LaeA	0.48	0.018

Table S2. Oligonucleotides used in this study.

Name	Sequence (5'→3')a	Purpose
oligo109	GCAATGTAAAGCTAACGTGCGTG	5' *AnipyrG* marker
oligo110	TGCCTTTAAGCTTCGGGTAGAG	3' *AnipyrG* marker
oligo386	TAGGCGGCTCCAGAGTCTTA	5' flanking region of *mbsA*
oligo1179	*TTTGTAGGCTTTGGGCTGTTCACAA*GCCGTGGAGGATGATAA	5' *mbsA* with *AnipyrG* tail
oligo1180	TTG	3' *mbsA* with *AnipyrG* tail
oligo389	*CTGATCTACCCCTTGGAACGCAGCA*TTTTCATGTCTTTGCGCA	3' flanking region of *mbsA*
oligo390	TC	5' nested of *mbsA*
oligo391	GCATTCCGCAATTGAGACTT	3' nested of *mbsA*
oligo699	GCATTCCGCAATTGAGACTT	5' *ef1α* for RT-qPCR normalization
oligo700	TGTTGCGACTCATGACCTGG	3' *ef1α* for RT-qPCR normalization
oligo256	CCATGTGTGTCGAGTCCTTC	5' *brlA* for RT-qPCR
oligo257	GAACGTACAGCAACAGTCTGG	3' *brlA* for RT-qPCR
oligo303	TTCCAAGCAGAGCTTGTCAC	5' *abaA* for RT-qPCR
oligo304	CCAGGTTCTTTGCACTTGAA	3' *abaA* for RT-qPCR
oligo305	GCTACCACTCTGCATCCTCA	5' *wetA* for RT-qPCR
oligo306	TACGAGCTCCAGCATGATTC	3' *wetA* for RT-qPCR
oligo689	ACGGCAGGAAGTTGTCTTCT	5' *gliP* for RT-qPCR
oligo690	CTGTCAGCGACTTGTTGGAT	3' *gliP* for RT-qPCR
oligo691	AAACCCCTGTGAATGCAGAC	5' *gliM* for RT-qPCR
oligo692	CCCCTTGAGATGAAAGGTGA	3' *gliM* for RT-qPCR
oligo693	CGATCTGTACCCCAACGAGT	5' *gliT* for RT-qPCR
oligo694	TTCTGGAACTTTGCCAGCTT	3' *gliT* for RT-qPCR
oligo1264	ACTCCACCATCCAGTTCCAG	5' *gliA* for RT-qPCR
oligo1265	TCCGAGTATCCCTCGATGTC	3' *gliA* for RT-qPCR
oligo1086	ACGGTGGCGGATATCGGGTGGTA	5' *mbsA* for RT-qPCR
oligo1087	AAGACGAGGACGGCGGCGAGAAAG	3' *mbsA* for RT-qPCR
oligo258	CTCGGAAGCGTATCAGGTCGTCT	5' *rodA* for RT-qPCR
oligo259	GGTTCCGTCGGGGTAGGCTCTT	3' *rodA* for RT-qPCR
oligo269	CTCTTCAACCAGTGCTCCAA	5' *rodB* for RT-qPCR
oligo270	GCAATGTTCTGCTTGCACTT	3' *rodB* for RT-qPCR
oligo1274	TGAAGTTCCTCGCTGTTGTC	5' *rodC* for RT-qPCR
oligo1275	GTGTTCACCGCACTTGTTCT	3' *rodC* for RT-qPCR
oligo418	GATGCGCAAGTCCCTGTGGTC	5' *mpkA* for RT-qPCR
oligo419	ACGCATCGCCGGGGTTCTTCT	3' *mpkA* for RT-qPCR
oligo426	CGGCTACATGACCGAATATG	5' *rlmA* for RT-qPCR
oligo427	ACACGTCGATAGCTTTGGTG	3' *rlmA* for RT-qPCR

oligo508	CAGCTCCTGATATCAAGCCA	5' *sakA* for RT-qPCR
oligo509	CGCTTGGTATTTGCACCTTA	3' *sakA* for RT-qPCR
oligo1310	GAGAATACCCTGCGGTTTGT	5' *pbsB* for RT-qPCR
oligo1311	GGATCCTCTTCTTGGGATCA	3' *pbsB* for RT-qPCR
oligo1312	GGGCAAGAATCGGTCGCAGAAGTT	5' *sskB* for RT-qPCR
oligo1313	CGCAGGGATGGCACCGAAGTAAGA	3' *sskB* for RT-qPCR
oligo1182	CGCGGCGATACTTCCAGGTT	5' *chsC* for RT-qPCR
oligo1183	CATAATCGCCCACTCGTTGTCTA	3' *chsC* for RT-qPCR
oligo1184	GGCAACTTCCTCTCCTCCTT	5' *chsA* for RT-qPCR
oligo1185	CCAGGAACTGGTCAGGATGT	3' *chsA* for RT-qPCR
oligo1186	CCAACGTACATCAACGTGCT	5' *cshB* for RT-qPCR
oligo1187	TTGGCCAGTTCTGCCTCATA	3' *chsB* for RT-qPCR
oligo1262	CAAGGACTCTGAACGCACTG	5' *cshE* for RT-qPCR
oligo1263	CCCTTCTCTGCTCCCTTGAA	3' *chsE* for RT-qPCR
oligo1422	ATCGCTGCATTCCTGGGTCTGTCG	5' *hygB*
	CGCGCAAGCTCATCGGCATTTT	
oligo1423	CCGGCTCGGTAACAGAACTANNNNNNNNNNGCCNNN	3' *hygB*
	NNNNNNNCAGAACGGCGTAACCAAAAGTCAC	
oligo1525	GTTGGAGCATATCGTTCAGAGCNNNNNNNNNNTAGN	3' *mbsA* with *hygB* tail for
oligo1526	NNNNNNNNNTTCATCTTGACGACCGTTGATCTG	complementation
	*TAGTTCTGTTACCGAGCCGG*GGCTGATGCGCAAAGACATG	5' *mbsA* with *hyg*B tail for
	*GCTCTGAACGATATGCTCCAAC*CATCTCTCGGAGTTGTCACA	complementation

[a] Tail sequence is in italic.
